# Mechanistic inhibition of herpes simplex virus-1 UL21 immune-evasion function by natural-product scaffolds: A multi-tier docking, dynamics, and energetic profiling approach

**DOI:** 10.1371/journal.pone.0355499

**Published:** 2026-09-16

**Authors:** Muhammad Suleman, Imtiaz Ali, Abrar Mohammad Sayaf, Fakhrul Hassan, Usama Ilahi, Mohammed Alissa, Sergio Crovella, Abdullah A. Shaito, Hadi M. Yassine

**Affiliations:** 1 Biomedical Research Center (BRC), QU Health, Qatar University, Doha, Qatar; 2 Center for Biotechnology and Microbiology, University of Swat, Swat, Pakistan; 3 School of Chemical Sciences, Universiti Sains Malaysia, Gelugor, Penang, Malaysia; 4 Department of Medical Laboratory Technology, Faculty of Rehabilitation and Allied Health Sciences, Riphah International University, Islamabad, Pakistan; 5 Department of Medical Laboratory, College of Applied Medical Sciences, Prince Sattam bin Abdulaziz University, Al-Kharj, Saudi Arabia; 6 Department of Biomedical Sciences, College of Health Sciences, QU Health, Qatar University, Doha, Qatar; AMET University, INDIA

## Abstract

Alpha-herpesviruses, particularly herpes simplex virus type 1 (HSV-1), establish lifelong latency and employ multiple strategies to evade host immunity, thereby sustaining infection. A key mediator of this immune evasion is the viral tegument protein, unique long 21 (UL21), which disrupts the host cyclic GMP-AMP synthase-stimulator of interferon genes (cGAS-STING) signaling pathway and suppresses antiviral type-I interferon responses, facilitating viral replication. Despite its important role in HSV-1 pathogenesis, UL21 remains an underexplored therapeutic target. This study aimed to identify natural-product scaffolds capable of targeting the UL21 N-terminal domain and potentially interfering with UL21-mediated immune evasion. Using comprehensive virtual screening of East and South African natural-product libraries, we employed a multi-tier computational workflow comprising molecular docking, molecular dynamics (MD) simulations, and binding free-energy calculations. Four compounds, Saundersioside C, kaempferol 3,7,4′-tri-O-β-glucoside, soyasaponin II, and OSW‑1, emerged as promising UL21-binding candidates with favorable docking scores and stable interaction profiles. Docking scores for saundersioside C, kaempferol 3,7,4′-tri-O-β-glucoside, soyasaponin II, and OSW-1 were −9.05, −8.94 kcal/mol, −7.45 kcal/mol, and −7.30 kcal/mol, respectively. Molecular dynamics (MD) trajectory analyses demonstrated stable conformational behavior as indicated by consistent root mean square deviation (RMSD), limited structural fluctuations, and persistent structural compactness. Total binding free-energy calculations further identified kaempferol 3,7,4′-tri-O-β-glucoside (−40.9 kcal/mol MM/GBSA; −33.9 kcal/mol MM/PBSA) as the compound with the most favorable predicted binding affinity toward UL21. Collectively, these findings identify natural-product scaffolds with high potential to modulate UL21-mediated immune suppression, with kaempferol 3,7,4′-tri-O-β-glucoside emerging as the most promising candidate. The findings provide a foundation for experimental validation and future antiviral drug development targeting HSV-1.

## Introduction

Alpha-herpesviruses, members of the *Herpesviridae* family, are characterized by rapid replication, a broad host range, and the ability to establish lifelong latency in sensory neurons. These viruses primarily replicate in epithelial and mucosal tissues and can periodically reactivate, leading to recurrent infections [1]. The major human alpha-herpesviruses include herpes simplex virus types 1 and 2 (HSV-1 and HSV-2) and varicella-zoster virus (VZV), which together represent a clinically important group of neurotropic double-stranded DNA viruses [2]. Globally, HSV-1 affects nearly 3.78 billion people aged 0–49 years, while genital HSV-1 affects about 376 million individuals aged 15–49 years. HSV-2 remains a major cause of genital herpes, with approximately 519.5 million prevalent cases and 25.6 million new infections annually worldwide [3].

Most HSV infections are asymptomatic, but symptomatic cases can present with vesicular or ulcerative lesions on the skin and mucous membranes. Recurrence results from viral reactivation in sensory neurons, often triggered by stress, fever, or immunosuppression, followed by spread along nerve fibers to peripheral tissues. Although both HSV-1 and HSV-2 establish persistent infections, HSV-2 is generally associated with more frequent and severe recurrences. Less common complications include herpes keratitis, Bell’s palsy, eczema herpeticum, and herpes gladiatorum [4–6]. HSV-1 is primarily transmitted through oral contact and is commonly associated with oral and facial herpes, whereas HSV-2 is mainly linked to genital herpes transmitted through sexual contact [7]. In recent decades, increased oral-genital transmission has contributed to a rise in genital HSV-1 infections; however, recurrence rates remain lower than those associated with HSV-2 [8].

HSV encodes a protease enzyme that plays a critical role in viral assembly and maturation by cleaving immature capsid proteins and promoting the structural remodeling required for nucleocapsid formation [9]. Following proteolytic processing, mature capsid proteins assemble with viral DNA to generate the nucleocapsid, which is subsequently enveloped by a lipid membrane to form a fully infectious viral particle [10]. Alpha-herpesviruses encode approximately eight capsid proteins, twenty-three tegument proteins, and fourteen envelope proteins [11,12]. Among these, the tegument layer, located between the capsid and envelope, performs essential functions during infection, including viral entry, secondary envelopment, and nuclear transport of the viral capsid [13].

The α-herpesvirus tegument protein UL21 has been identified as a multifunctional factor that facilitates efficient viral replication while modulating host innate immune responses. UL21 disrupts activation of the cGAS–STING signaling pathway, a key cytosolic DNA-sensing mechanism, leading to reduced type-I interferon production and enabling viral replication to progress with minimal antiviral interference [14]. In HSV-1, UL21 also contributes to infectious virus production by supporting cell-to-cell spread and promoting early events in the viral life cycle [15,16]. In addition, UL21 functions as an adaptor for protein phosphatase 1, thereby further supporting herpesvirus replication and dissemination [17]. Recent mechanistic studies have shown that the N-terminal domain of UL21 promotes Toll-interacting protein (TOLLIP)-dependent selective autophagy through ubiquitin protein ligase E3C (UBE3C)-mediated K27-linked ubiquitination of cGAS, leading to cGAS degradation and suppression of innate immune signaling [18,19]. Therefore, the N-terminal domain of UL21 represents a structurally and biologically relevant site for therapeutic intervention.

Despite major advances in antiviral therapy, strategies that directly target HSV-1 immune-evasion mechanisms remain limited [16]. In particular, UL21 remains an attractive yet underexplored antiviral target, with no specific inhibitors currently available to disrupt its immunosuppressive function, highlighting a clear gap in current HSV-1 antiviral strategies. Natural products have historically served as a rich source of structurally diverse and biologically active compounds, offering significant potential for the discovery of novel antiviral agents [20–22]. In this regard, the African natural-product libraries represent a largely untapped resource of unique chemical scaffolds with promising pharmacological properties [23–25].

**Fig 1:**
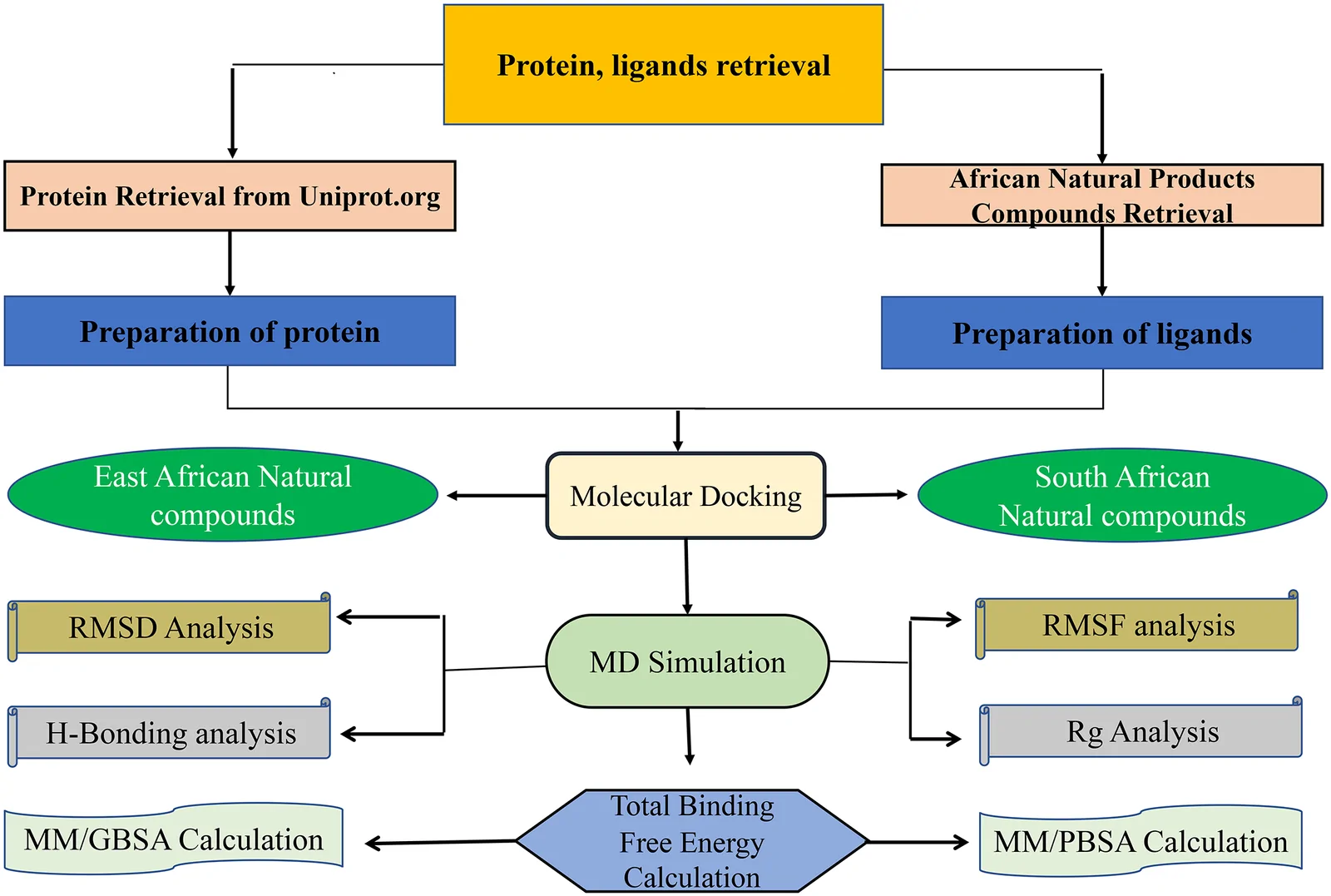
Schematic workflow of the study illustrating the integrated computational pipeline employed in this work. The workflow includes virtual screening and molecular docking of selected ligands against the UL21 protein, followed by molecular dynamics (MD) simulations to evaluate the stability and dynamic behavior of the protein-ligand complexes. Finally, binding free energy calculations were performed to assess the binding affinity and overall interaction strength of the selected compounds.

Advances in computational drug discovery, including virtual screening, molecular docking, induced-fit docking, molecular dynamics simulations, and binding free-energy analysis, provide an efficient strategy for identifying and characterizing candidate inhibitors against challenging viral targets [26]. Accordingly, the present study systematically investigated East and South African natural-product databases against the N-terminal domain of HSV-1 UL21. The study integrated molecular docking-based virtual screening with induced-fit refinement to evaluate binding affinity, interaction profiling to identify critical ligand–residue interactions, molecular dynamics simulations alongside binding free-energy calculations to assess complex stability, and ADMET analysis to determine drug-likeness and pharmacokinetic characteristics (Figure 1). This integrated framework prioritized natural-product scaffolds with favorable binding, dynamic stability, and drug-development potential that may interfere with UL21-mediated immune evasion and support restoration of host antiviral defense mechanisms.

## Materials and methods

### Crystal structure retrieval and preparation

The crystal structure of UL21 N-terminal Domain of HSV-1 (PDB ID: 4U4H) was retrieved from Research Collaboratory for Structural Bioinformatics, Protein Data Bank (RCSB, PDB) (https://doi.org/10.2210/pdb4U4H/pdb) [27]. The structure was then subjected to PyMOL for the removal of water molecules [28]. The addition of hydrogen atoms and minimization of the protein structure was achieved by using Chimera [29,30]. Further, Ionizable residues were assigned their standard protonation states at physiological pH (7.4), ensuring chemically consistent structures suitable for docking.

### Retrieval and preparation of natural products libraries

The natural-product libraries obtained from EANPDB (1,871 compounds) and SANCDB (1,012 compounds) were collected, standardized, and prepared for virtual screening. These databases comprise structurally diverse natural compounds derived from African flora and fauna, many of which have been previously recognized for their medicinal and pharmacological potential [23,25,31]. Before performing molecular docking analysis with EasyDock Vina 2.0, all ligands were transformed into the PDBQT format [32]. Ligand preparation was carried out using Open Babel, which was employed to assign atomic charges and atom types. During this process, ionizable groups were assigned appropriate protonation states corresponding to physiological pH (7.4) to ensure chemically accurate and biologically relevant structures. During this procedure, non-polar hydrogen atoms were merged, Gasteiger partial charges were assigned, and torsion tree roots were defined to account for ligand flexibility, ensuring that all compounds were properly optimized for subsequent docking simulations.

### Grid box generation

The online PrankWeb Ligand Binding Site Prediction tool (https://prankweb.cz/) was used to identify the putative ligand binding site and determine the grid box center for molecular docking [33]. The top ranked predicted pocket, Rank 1, had a pocket score of 5.51 and a binding probability of 0.277. It comprised residues: A_71, A_75, A_78, A_79, A_82, A_83, A_96, A_98, A_116, A_117, A_118, and A_122. The coordinates of the predicted pocket center were X = −82.70, Y = −4.38, and Z = 22.29. The pocket had an average evolutionary conservation score of 2.62, indicating moderate conservation.

To further validate the predicted binding region, the protein structure was independently analyzed using CavityPlus (http://www.pkumdl.cn:8000/cavityplus/) [34] and DoGSiteScorer through the ProteinsPlus server (https://proteins.plus/) [35].

CavityPlus identified the highest ranked cavity, comprising residues: A_66, A_67, A_68, A_71, A_72, A_81, A_82, A_83, A_84, A_85, A_86, A_87, A_88, A_93, A_94, A_97, A_98, A_99, A_100, A_101, A_102, A_103, A_104, A_107, and A_121. The cavity had a predicted maximum pKd of 9.72 and a predicted average pKd of 5.95, supporting its potential ligandability. DoGSiteScorer independently identified the highest ranked pocket, composed of residues: A_28, A_30, A_31, A_35, A_47, A_73, A_74, A_75, A_76, A_77, A_78, A_79, A_81, A_82, A_84, A_85, A_90, A_92, A_93, A_96, A_97, A_165, A_167, A_170, A_171, and A_174. Although both tools identified broader pocket boundaries, their predictions overlapped with the region selected by PrankWeb. Overall, these independent predictions support the selection of the binding site used to define the molecular docking grid.

Importantly, the selected binding region lies within the conserved N-terminal domain of UL21, which adopts a distinctive sail-like fold and contains evolutionarily conserved surface patches of potential functional and therapeutic relevance [27]. Relatedly, this domain contributes to HSV-1 immune evasion function by promoting UBE3C-mediated ubiquitination and TOLLIP-mediated selective autophagic degradation of cGAS, thereby suppressing innate immune and interferon responses [18]. These structural and functional features support the UL21 N-terminal domain as a biologically relevant antiviral target.

### Virtual screening and induced-fit docking

The AutoDock Vina was used for virtual screening to assess and prioritize candidate compounds. Initial screening was performed at an exhaustiveness level of 16 to rapidly evaluate very large libraries. Subsequently, the most promising compounds were re-screened using a higher exhaustiveness value of 64 to minimize false positives and improve hit selection accuracy. Compounds were ranked based on their binding affinity scores, and the top 10% of compounds from each database, which exhibited docking scores in the range of −6 to −8 kcal/mol, were selected for further analysis. Specifically, 187 compounds from EANPDB and 100 compounds from SANCDB met this selection criterion and were subsequently subjected to induced-fit docking (IFD) using AutoDockFR. This cutoff strategy was applied to reduce computational complexity while retaining structurally diverse and energetically favorable ligands for detailed investigation. AutoDockFR applies force-field-based scoring functions and simulation protocols like molecular dynamics to manage receptor flexibility. The default IFD (Induced-Fit Docking) parameters were used which allowed for adjustments of flexible-receptors and covalent docking support thus increasing the accuracy and reliability of the binding predictions [36]. The visualization and analysis of complexes obtained from induced-fit docking (IFD) were carried out using PyMOL and the freely available academic version of Schrödinger Maestro, enabling the assessment of binding orientations and key molecular interactions. The four top-ranking hits were selected and subsequently subjected to molecular dynamics (MD) simulations for further evaluation and validation.

### Molecular dynamics simulation of top-scoring hits

Molecular dynamics (MD) simulations for the top docked ligand–protein complexes were carried out using the Amber21 package. System preparation was performed using tLeap, where topology and coordinate files were generated. Each complex was solvated in an OPC (Optimal Point Charge) water model within a truncated octahedral box, maintaining a minimum distance of 10 Å [37]. Counterions (Na^+^/Cl^-^) were added to neutralize the system. Ligand parameters were generated using the GAFF2 (General AMBER Force Field 2), while atomic partial charges were assigned using the AM1-BCC method via Antechamber. Missing parameters were corrected using Parmchk2 [38,39]. The protein was described using the ff19SB force field. Energy minimization was performed in two stages: (i) initial minimization of solvent and ions with positional restraints on the solute for 5,000 steps (2,500 steepest descent followed by 2,500 conjugate gradient), and (ii) unrestrained minimization of the entire system for 10,000 steps [40]. Following minimization, the system was gradually heated from 0 K to 300 K over 200 ps under constant volume (NVT) conditions using a Langevin thermostat with a collision frequency of 1 ps^-1^. Subsequently, equilibration was performed under constant pressure (NPT) conditions at 1 atm and 300 K for 1 ns using the Berendsen barostat with a pressure relaxation time of 2 ps [41]. Long-range electrostatic interactions were treated using the Particle Mesh Ewald (PME) method with a non-bonded cutoff of 10 Å [42]. The Lennard-Jones potential was applied to describe van der Waals interactions. The SHAKE algorithm was used to constrain all bonds involving hydrogen atoms, allowing a time step of 2 fs. After equilibration, a 100-ns production simulation was carried out under NPT conditions (300 K, 1 atm) to maintain a stable and realistic physiological environment [43]. Trajectory analysis was performed using the CPPTRAJ module. Root mean square deviation (RMSD), root mean square fluctuation (RMSF), and Radius of gyration (Rg) were calculated using frames extracted at 10 ps intervals from the full production trajectory.

### Post-simulation analysis of the top hits-UL21 complexes

The trajectory obtained from the simulation production was examined through the use of CPPTRAJ module [44]. Several key metrics, including RMSD, RMSF, SASA, and hydrogen bonding were computed for each system during this analysis [45–47]. The total post-simulation hydrogen bonds network within the simulated system was conducted using a donor-acceptor distance cutoff of 3.5 Å and an angle cutoff of 135°. RMSD, a measure of the difference between the original and superimposed structures, is obtained by means of the following equation:


$$\begin{array}{c} RMSD=\sqrt{\frac{\text{1}}{N}\sum_{i=\text{1}}^{N}\;\delta_{i}^{\text{2}}} \end{array}$$ (i)

N represents the total number of atoms, δᵢ is the distance between atom i and the reference structure.

RMSF measures the local flexibility of individual amino acid residues over the course of the simulation trajectory. It calculates the average displacement of individual residues from their mean reference positions, with the corresponding values calculated by means of the following equation:


$$\begin{array}{c} RMSF=\sqrt{\frac{\text{3}B}{\text{8}\pi^{\text{2}}}} \end{array}$$ (ii)

where B represents the B-factor (temperature factor from crystallography). The B-factor reflects the mean-square displacement of an atom around its equilibrium position and is expressed as:


$$\begin{array}{c} B=\frac{\text{8}\pi^{\text{2}}}{\text{3}}\left\langle \Delta r^{\text{2}}\right\rangle \end{array}$$ (iii)

where ⟨Δr²⟩ represents the mean-square atomic displacement. r_RG_ is the radius of gyration and r^2^_RG_ functions as an indicator of the overall structural compactness and structural integrity of the protein-ligand complex over the simulation trajectory:


$$\begin{array}{c} r_{\text{RG}}^{\text{2}}=\frac{\sum_{i=\text{1}}^{N}\;m_{i}{\left({𝐫}_{i}-{𝐫}_{\text{CM}}\right)}^{\text{2}}}{\sum_{i=\text{1}}^{N}\;m_{i}} \end{array}$$ (iv)

mᵢ denotes the mass of atom i, rᵢ is the position vector of atom i, and rCM represents the coordinates of the center of mass of the protein.

### Binding free energy calculation of lead compounds-UL21 complexes

Binding free energy analysis is a key method for evaluating ligand–protein interactions, as it quantifies both the energetic contribution and the binding strength between interacting molecules. More negative (lower) binding free energy values indicate stronger binding affinity, thereby aiding ligand selection and validation of docking results [48]. Consequently, the binding affinities of selected African natural compounds with the UL21 protein were determined utilizing the Molecular Mechanics/Generalized Born Surface Area (MM/GBSA) method. 500 representative frames extracted from the 100 ns MD trajectory at an interval spacing of 10 frames using MMPBSA.py module in Amber21 [49].

The following mathematical equations are used to calculate the binding free energies:


$$\begin{array}{c} {\Delta G}_{bind}=\;G_{\left(complex,\;solvated\right)}-\;G_{\left(ligand,\;solvated\right)}-\;G_{\left(receptors,\;solvated\right)} \end{array}$$ (v)

Each component contributing to the total binding energy was calculated by the following equation:


$$\begin{array}{c} G=\;E_{Molecular\;Mechanics}-\;G_{solvated}-\;TS \end{array}$$ (vi)

$$\begin{array}{c} {\Delta G}_{bind}=\;{\Delta E}_{Molecular\;Mechanics}+\;{\Delta G}_{solvated}-\;\Delta TS=\;{\Delta G}_{vaccum}\;+\;{\Delta G}_{solvated} \end{array}$$ (vii)

$$\begin{array}{c} {\Delta E}_{Molecular\;Mechanics}=\;{\Delta E}_{int}+\;{\Delta E}_{electrostatic}\;+\;{\Delta E}_{vdW} \end{array}$$ (viii)

$$\begin{array}{c} \Delta G_{solvated}=\;\Delta G_{Generalized\;born}+\Delta G_{surface\;area} \end{array}$$ (ix)

$$\begin{array}{c} \Delta G_{surface\;area}=\;\gamma .SASA+b \end{array}$$ (x)

$$\begin{array}{c} \Delta G_{vaccum}=\;{\Delta E}_{Molecular\;Mechanics}-T\Delta S \end{array}$$ (xi)

### PCA (Principal Component Analysis) and FEL (Free Energy Landscape) analysis of lead compounds-UL21 complexes

PCA was applied to the equilibrated MD trajectories using CPPTRAJ to capture the major collective motions of the complexes. After removing solvent and ions, the trajectories were re-centered, imaged, and RMS-aligned to a reference frame. A covariance matrix built from Cα atomic fluctuations was diagonalized to obtain eigenvectors representing the dominant motion modes. The trajectory was then projected onto PC1 and PC2 to visualize conformational sampling. These projections were subsequently used to construct the free energy landscape (FEL), where the relative free-energy states were calculated through the Boltzmann relationship to identify stable conformational basins.

### Pharmacokinetics analysis of selected compounds

The pharmacokinetic profile of the selected compounds was assessed using an in silico ADMET approach. First, their SMILES notations were obtained from the PubChem database. These molecular representations were then analyzed through the pkCSM platform (http://biosig.unimelb.edu.au/pkcsm/) to predict key absorption, distribution, metabolism, excretion, and toxicity parameters. The evaluation included important indicators such as human intestinal absorption efficiency, Caco-2 cell permeability, and aqueous solubility (expressed as log mol/L). Distribution-related properties were examined by estimating blood–brain barrier penetration, using standard logBB thresholds to determine CNS accessibility. Furthermore, metabolic behavior was characterized by identifying interactions with cytochrome P450 enzymes, including both substrate specificity and inhibitory potential. Toxicological endpoints were also considered, including AMES mutagenicity, along with assessments of skin sensitization and hepatotoxicity, to ensure a comprehensive safety evaluation of the compounds [50].

## Results and discussion

### Virtual screening and docking analysis

**Fig 2:**
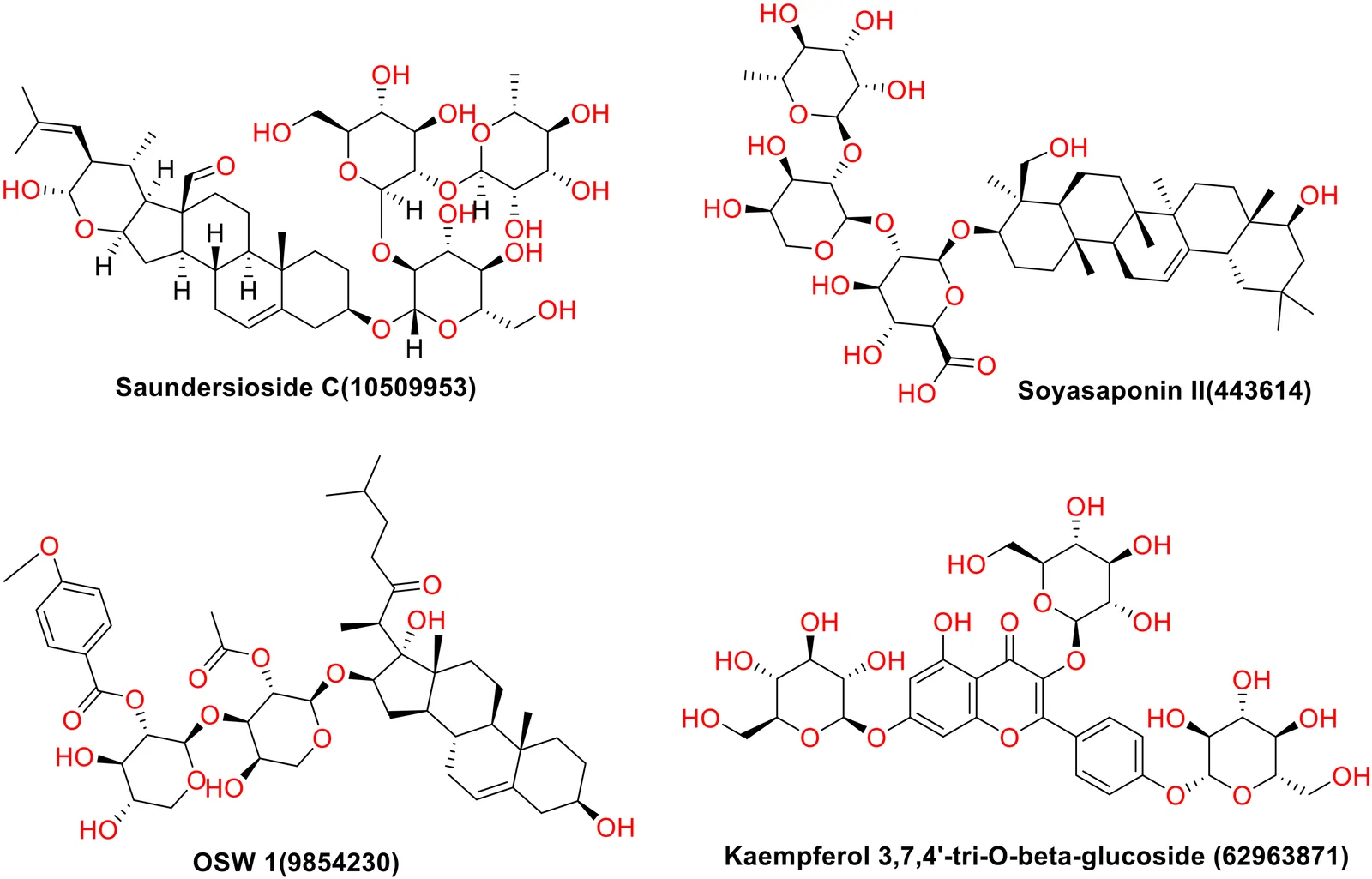
Two-dimensional chemical structures of the top four hit compounds identified through virtual screening against the N-terminal domain of the UL21 protein.

Natural compounds have long served as a rich source of bioactive molecules with significant therapeutic potential, particularly in the discovery of antiviral agents targeting complex viral mechanisms. In the present study, we explored the potential of African natural compounds to inhibit the UL21-mediated immune evasion mechanism in HSV-1 through molecular docking and virtual screening approaches. After the UL21 protein preparation, molecular docking was carried out using AutoDock Vina, which is a reliable computational tool for the prediction of ligand-protein interactions and their binding affinities. The databases of African natural products were screened for the N-terminal domain (NTD) of UL21. The Protein-Ligand Interaction Profiler (PLIP) was used to analyze and visualize the interactions between the identified compounds and UL21 [51]. The induced-fit docking identified four top hit compounds based on highest docking scores. Among the East African compounds, Kaempferol 3,7,4’-tri-O-β-glucoside had the best performance with a docking score of −8.94 kcal/mol and formed twelve hydrogen bonds. On the other hand, Soyasaponin II scored −7.45 kcal/mol and formed seven hydrogen bonds. In the case of the South African database, Saundersioside C revealed the highest docking score of −9.05 kcal/mol and thirteen hydrogen bonds, while OSW-1 got a score of −7.30 kcal/mol and had twelve hydrogen bonds. Fig 2 depicts the chemical structures, and Table 1 presents the detailed binding interactions of these leading candidates.

**Table 1:** *List of top hit compounds identified from African natural product libraries, along with their docking scores and key interaction details with the N-terminal domain of* UL*21.*

Compounds	Docking Score	Ligand FG/atom	Residue	Distance (Å)	Interaction Nature
Saundersioside C (CID:10509953) SANPDB	−9.1	OH(Pyrandiol)	Tyr75	1.85	HB
		OH(Pyrandiol)	Glu79	2.06	HB
		OH(Pyrandiol)	Glu79	2.84	HB
		OH(Pyrandiol)	Arg83	3.17	HB
		OH(Pyrantriol)	Leu115	3.25	HB
		OH(Pyrantriol)	Leu115	1.78	HB
		OH(Pyrandiol)	Asp116	2.29	HB
		O(ether)	Glu117	3.58	HB
		OH(Pyrandiol)	Glu117	2.02	HB
		OH(Pyrandiol)	Glu117	1.98	HB
		O(ether)	Cys118	2.97	HB
		OH(Pyrandiol)	Glu121	1.75	HB
		OH(Pyrandiol)	Arg127	2.16	HB
		Cyclo pentane ring	Val71	3.77	HI
		2-methylbut-2-ene	Tyr75	3.45	HI
		Cylco hexane ring	Tyr75	3.47	HI
		Cylco hexane ring	Tyr122	3.91	HI
kaempferol 3,7,4’-tri-O-beta-glucoside (CID:162963871) EANPDB	−8.9	OH-CH2(Pyran-3,4,5-triol)	Thr65	2.06	HB
		OH-CH2(Pyran-3,4,5-triol)	Tyr75	1.78	HB
		OH(Pyran-3,4,5-triol)	Ser78	2.09	HB
		OH(Pyran-3,4,5-triol)	Ser78	2.53	HB
		OH(Pyran-3,4,5-triol)	Glu79	3.18	HB
		OH(Pyran-3,4,5-triol)	Glu79	1.82	HB
		OH(Pyran-3,4,5-triol)	Gln82	3.00	HB
		OH(Pyran-3,4,5-triol)	Arg83	2.89	HB
		OH-CH2(Pyran-3,4,5-triol)	Cys118	2.24	HB
		OH(Pyran-3,4,5-triol)	Glu121	1.93	HB
		OH(Pyran-3,4,5-triol)	Glu121	3.21	HB
		OH(Pyran-3,4,5-triol)	Glu121	3.13	HB
		Benzene ring	Tyr75	3.27	HI
		Benzene ring	Tyr75	3.47	HI
		Benzene ring	Glu79	3.87	HI
		Phenol ring	Tyr122	3.94	HI
Soyasaponin II (CID:443614) EANPDB	−7.5	OH(dimethylcyclohexyl)methanol)	Tyr75	1.85	HB
		OH(cyclohexanol)	Ser78	1.93	HB
		OH(Pyran-3,4,5-triol)	Glu79	2.06	HB
		OH(Pyran-3,4,5-triol)	Glu79	2.84	HB
		OH(Pyran-3,4,5-triol)	Arg83	1.97	HB
		OH(Pyran-3,4,5-triol)	Arg83	2.73	HB
		OH(Pyran-3,4,5-triol)	Asp116	2.29	HB
		OH(Pyran-2-carboxylic acid)	Glu117	3.58	HB
		COOH(Pyran-2-carboxylic acid)	Glu117	2.02	HB
		OH(dimethylcyclohexyl)methanol)	Cys118	2.97	HB
		OH(Pyran-2-carboxylic acid)	Arg127	2.23	HB
		OH(Pyran-2-carboxylic acid)	Ser129	2.33	HB
		COOH(Pyran-2-carboxylic acid)	Arg127	2.16	SB
		CH3(cyclohexane)	Tyr75	3.45	HI
		CH3(cyclohexanol)	Tyr75	3.47	HI
OSW-1 (CID:9854230) SANPDB	−7.3	CO(Acetate)	Tyr75	2.13	HB
		OH(Pyran-3-yl acetate)	Glu79	1.69	HB
		OH(Pyran-3-yl acetate)	Arg83	2.21	HB
		OH(Pyran-3-yl acetate)	Arg83	2.64	HB
		OH(pyran-3,4-diol)	Asp116	1.98	HB
		OCH3(Anisole)	Arg127	2.92	HB
		OCH3(Anisole)	Arg127	1.89	HB
		Carboxylate	Arg83	3.28	SB
		Carboxylate	Arg83	4.96	SB
		Cyclohexanol	Tyr75	3.57	HI
		Cyclohexanol	Glu79	3.86	HI
		Anisole	Glu117	3.88	HI

**Fig 3:**
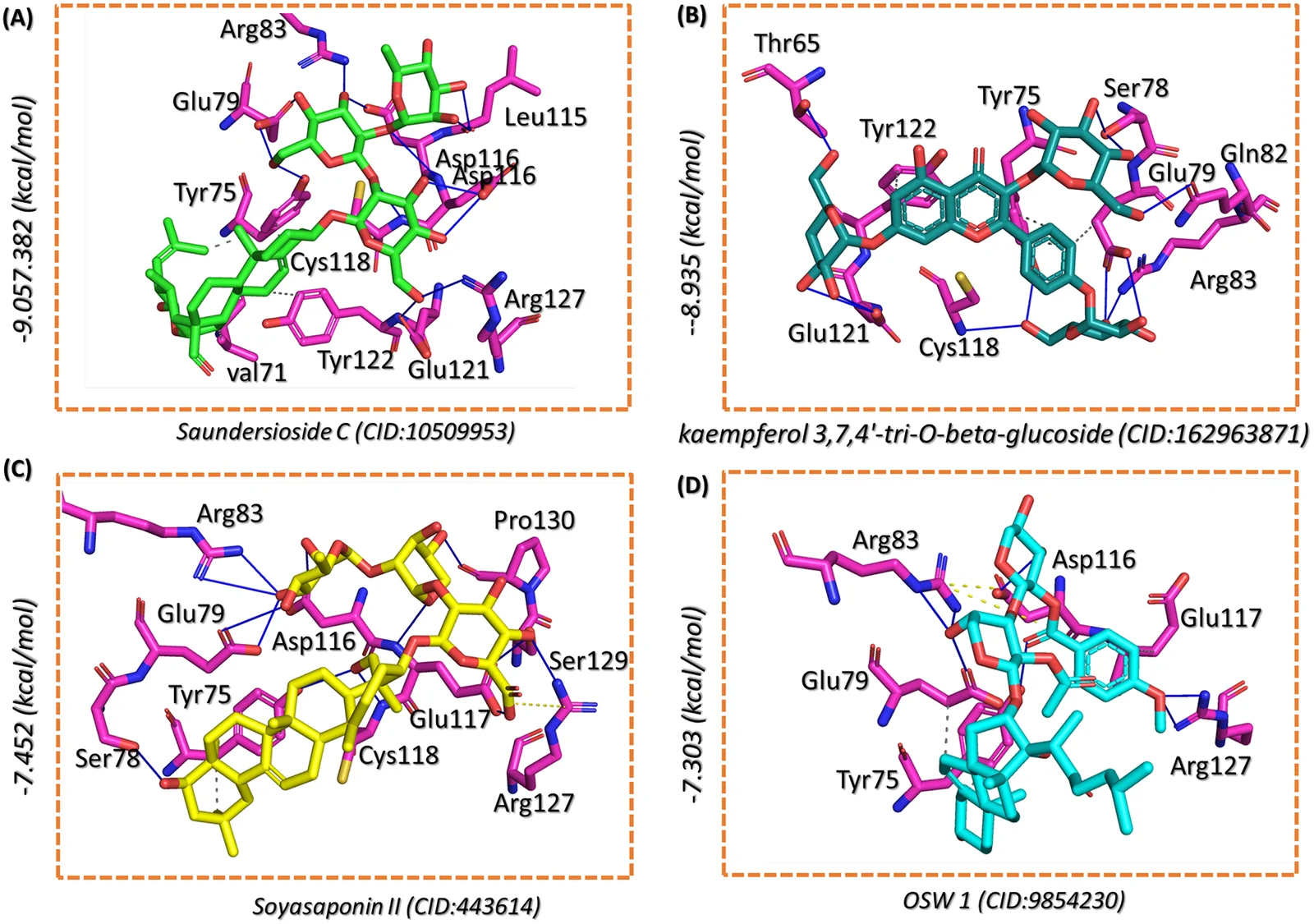
Interaction patterns of UL21 with the selected top-ranked ligands are illustrated using stick representations. (A) Binding interactions of UL21 with Saundersioside C. (B) Binding interactions of UL21 with kaempferol 3,7,4′-tri-O-β-glucoside. (C) Binding interactions of UL21 with Soyasaponin II. (D) Binding interactions of UL21 with OSW-1.

The Saundersioside C (CID:10509953) obtained from South African Natural Products Database (SANPDB), was found to have highest binding affinity with UL21 with the docking score of −9.1 kcal/mol indicates a very stable and favorable interaction. This compound has been cited in the literature as being able to inhibit the growth of leukemia HL-60 and MOLT-4 cell lines in vitro by **[**52**]**, thus reinforcing its probability of being a bioactive therapeutic candidate. Fig 3A depicts the results of the detailed interaction analysis which revealed that the compound established a large network of hydrogen bonds and made hydrophobic contacts with the active site of the UL21. Multiple hydroxyl groups from the pyrandiol and pyrantriol moieties formed key hydrogen bonds with several critical residues, including Tyr75 (1.85 Å), Glu79 (2.06 and 2.84 Å), Arg83 (3.17 Å), Leu115 (1.78 and 3.25 Å), Asp116 (2.29 Å), Glu117 (1.98, 2.02, and 3.58 Å), Cys118 (2.97 Å), Glu121 (1.75 Å), and Arg127 (2.16 Å), thereby stabilizing the ligand within the binding site through a dense network of polar interactions. In addition to these hydrogen bonds, several hydrophobic interactions (HI) further reinforced the ligand-protein complex stability. Specifically, the cyclopentane ring of Saundersioside C interacted with Val71 (3.77 Å), while the 2-methylbut-2-ene and cyclohexane rings formed hydrophobic contacts with Tyr75 (3.45 and 3.47 Å, respectively) and Tyr122 (3.91 Å).

Moving to Kaempferol 3,7,4’-tri-O-β-glucoside (CID:162963871), identified as the second top hit from the East African Natural Products Database (EANPDB), is a naturally occurring flavonoid glycoside isolated from *Warburgia ugandensis*, a medicinal plant known for its potent anti-inflammatory, antimicrobial, and anticancer properties [53]. With a docking score of −8.9 kcal/mol, this compound exhibited a strong and stable affinity for the UL21 N-terminal domain, suggesting its potential to interfere with UL21-mediated immune evasion. The detailed analysis as shown in Fig 3B indicated that there were numerous hydrogen bonds between the OH and OH-CH_2_ groups of the pyran-3,4,5-triol moieties and the important amino acid residues. Specifically, hydrogen bonds were observed with Thr65 (2.06 Å), Tyr75 (1.78 Å), Ser78 (2.09 and 2.53 Å), Glu79 (3.18 and 1.82 Å), Gln82 (3.00 Å), Arg83 (2.89 Å), Cys118 (2.24 Å), and Glu121 (1.93, 3.13, and 3.21 Å), establishing an extensive polar interaction network. Moreover, the interaction of the hydrophobic nature between the benzene and phenol rings of kaempferol and the residues Tyr75 (3.27 and 3.47 Å), Glu79 (3.87 Å), and Tyr122 (3.94 Å) contributed to the stabilization of the ligand in the UL21 binding pocket.

Similarly, Soyasaponin II (CID:443614), a bioactive saponin derived from soybean (*Glycine max*), emerged as the third top hit from the East African Natural Products Database (EANPDB), demonstrating a docking score of −7.5 kcal/mol and a strong binding affinity toward the UL21 N-terminal domain. This compound has been widely recognized for its anticancer and immunomodulatory properties [54]. Soyasaponin II established an extensive interaction network dominated by its numerous hydroxyl (-OH) and carboxyl (-COOH) functional groups, which contributed to a rich pattern of hydrogen bonds and electrostatic interactions. The hydroxyl groups from the dimethylcyclohexylmethanol, cyclohexanol, and pyran-3,4,5-triol moieties formed multiple hydrogen bonds with Tyr75 (1.85 Å), Ser78 (1.93 Å), Glu79 (2.06 and 2.84 Å), Arg83 (1.97 and 2.73 Å), and Asp116 (2.29 Å). Additional polar contacts were observed between the pyran-2-carboxylic acid group and residues Glu117 (2.02 and 3.58 Å), Cys118 (2.97 Å), Arg127 (2.23 Å), and Ser129 (2.33 Å). In addition to the polar interactions, non-polar contacts were found between the methyl groups (CH_3_) of the cyclohexane and cyclohexanol rings with Tyr75 (3.45 and 3.47 Å), which in turn helped the ligand to be stable in the UL21 binding cavity as illustrated in Fig 3C.

Additionally, OSW-1 (CID:9854230), the fourth highest hit from the SANPDB, is a powerful steroidal saponin obtained from Ornithogalum saundersiae. The compound exerts antitumor, cytostatic, and cytotoxic effects on HL-60 leukemia cells, as well as acting as a cyclic AMP phosphodiesterase inhibitor [55]. Having a docking score of −7.3 kcal/mol, OSW-1 exhibited a strong and stable binding affinity to UL21. Thorough analysis of the interactions showed that the carbonyl (CO) and the hydroxyl groups of the acetate part of pyran-3-yl acetate were interacting through hydrogen bonds with Tyr75 (2.13 Å), Glu79 (1.69 Å), and Arg83 (2.21 and 2.64 Å); the pyran-3,4-diol part of the molecule was involved in a hydrogen bond with Asp116 (1.98 Å). Moreover, the methoxy (OCH_3_) groups of the anisole ring were also contributing to the binding through interactions with Arg127 (1.89 and 2.92 Å). Additionally, two salt bridge interactions were observed between the carboxylate group of OSW-1 and Arg83 (3.28 and 4.96 Å), contributing to electrostatic stabilization. Multiple hydrophobic contacts involving the cyclohexanol ring and residues Tyr75 (3.57 Å), Glu79 (3.86 Å), and Glu117 (3.88 Å) further anchored the ligand within the binding pocket (Fig 3D).

### Dynamics stability analysis of the lead compounds-UL21 complexes

**Fig 4:**
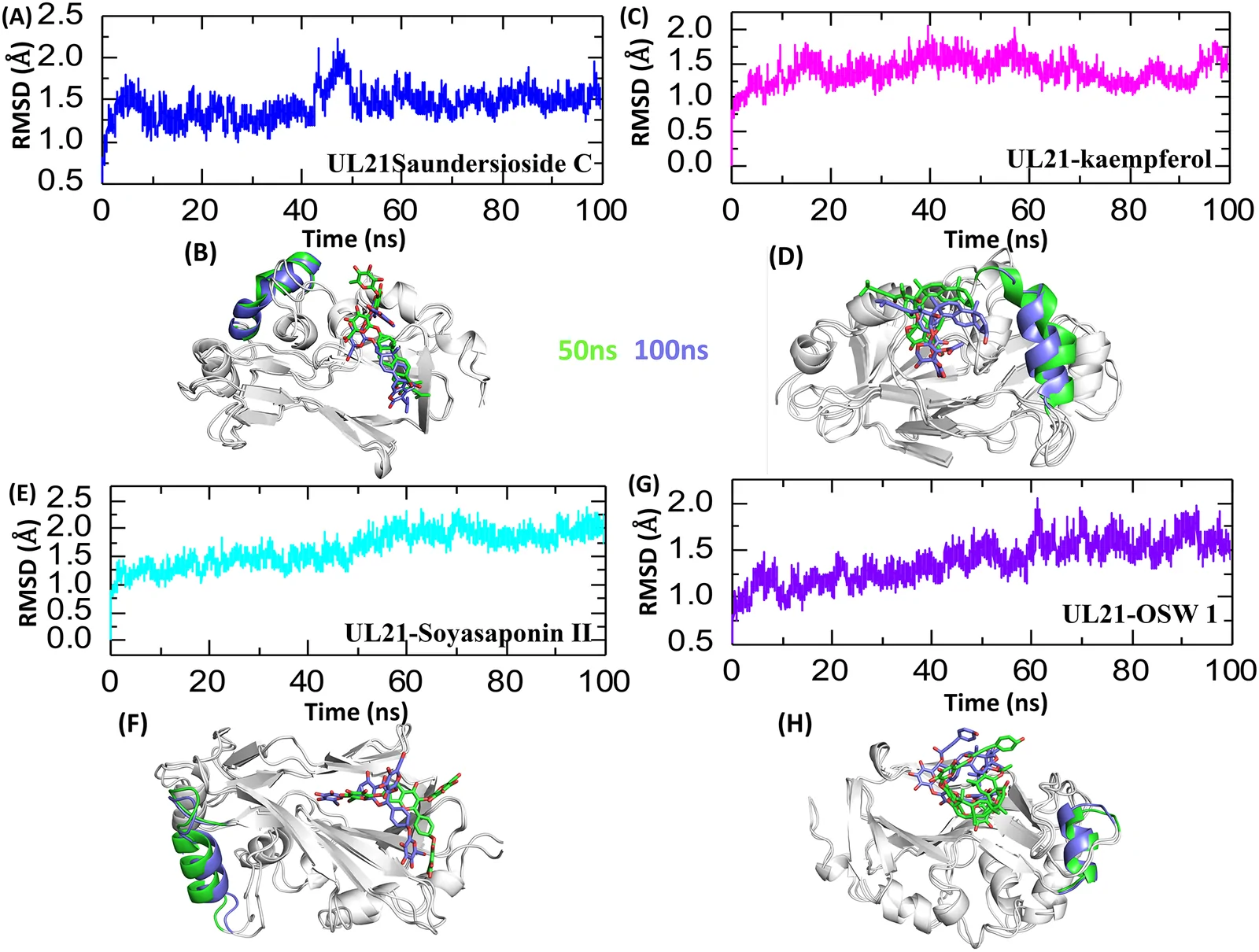
Post-simulation trajectory analysis of UL21-ligand complexes based on RMSD was performed to evaluate structural stability. (A, B) RMSD profile and stability analysis of the UL21-Saundersioside C complex. (C, D) Stability analysis of UL21-kaempferol complex as RMSD and post-simulation trajectories superimposition. (E, F) RMSD profile of the UL21-Soyasaponin II complex and post-simulation analysis. (G, H) RMSD profile of the UL21-OSW-1 complex during the 100 ns MD simulation.

Molecular dynamics (MD) simulations provide valuable insight into the time-dependent structural stability of protein-ligand complexes, offering a more realistic evaluation of binding behavior than static docking alone. Among the widely used MD descriptors, RMSD is a key parameter that measures the global conformational stability of a system by quantifying the deviation of the protein backbone from its initial structure over time. A constant RMSD profile strongly indicates that the complex has reached a very stable state and that the different molecules are consistently arranged at the site; hence, the pocket is able to accommodate the ligand reliably. Thus, RMSD trajectory analysis is important for affirming the dynamic stability of UL21 together with the chosen African natural products and for figuring out if these ligands have stable interactions throughout the simulation time. The RMSD analysis of the UL21-ligand complexes conducted over the 100-ns simulation clearly shows different behaviors in the structural stability and equilibration of each system, thereby supporting the docking results. All four complexes present a similar pattern in the initial rise of RMSD during the beginning of the simulation, which is an indication of the natural adjustment of the protein-ligand systems as they move from the docked conformations to the dynamically stable states (Fig 4). The UL21-Saundersioside C complex displayed RMSD values mainly in the range of 1.2 to 1.8 Å, which suggests a stable binding mode with only slight changes over the 50 ns period. The average RMSD for this complex was found to be 1.5 Å (Fig 4A). Moreover, the superimposition of the structures obtained from the simulation at different times (50 ns, and 100 ns) provided additional evidence for the strong positioning of the drug in the binding pocket, which in turn suggested the conformational stability of the complex over the whole simulation period (Fig 4B). In the same manner, the UL21-kaempferol complex demonstrates a stability of 1.4–1.7 Å after approximately 20 ns, thereby suggesting that the protein undergoes very small conformational changes and keeps a stable equilibrium during the production phase (Fig 4C). The overlay further affirms the idea of stable drug binding in the binding pocket of UL21 (Fig 4D). In the case of UL21-Soyasaponin II, there is a gradual RMSD increase that finally gets to around 2.0 Å at the simulation's middle point. However, it soon gets stabilized, which demonstrates that the system has come to equilibrium and that there has been no structural change in the last half of the trajectory. On the other hand, the average RMSD value is still higher than that of the first two complexes (Fig 4E). Furthermore, for the UL21-OSW-1 complex, a noticeable but isolated spike occurs near the 60 ns mark, which may represent transient conformational rearrangements; however, this is followed by stabilization with RMSD values clustering near 1.5 Å for the remainder of the run (Fig 4G). Moreover, the superimposition analysis showed that both Soyasaponin II and OSW-1 compounds stably bind in the binding cavity of UL21 protein (Fig 4F, 4H). Collectively, these RMSD profiles demonstrate that all complexes achieve stable dynamic behavior with no significant structural deviations, supporting the reliability of the predicted binding interactions and confirming that each ligand remains tightly associated with UL21 throughout the simulation.

### Root mean square fluctuations analysis of lead compounds-UL21 complexes

**Fig 5:**
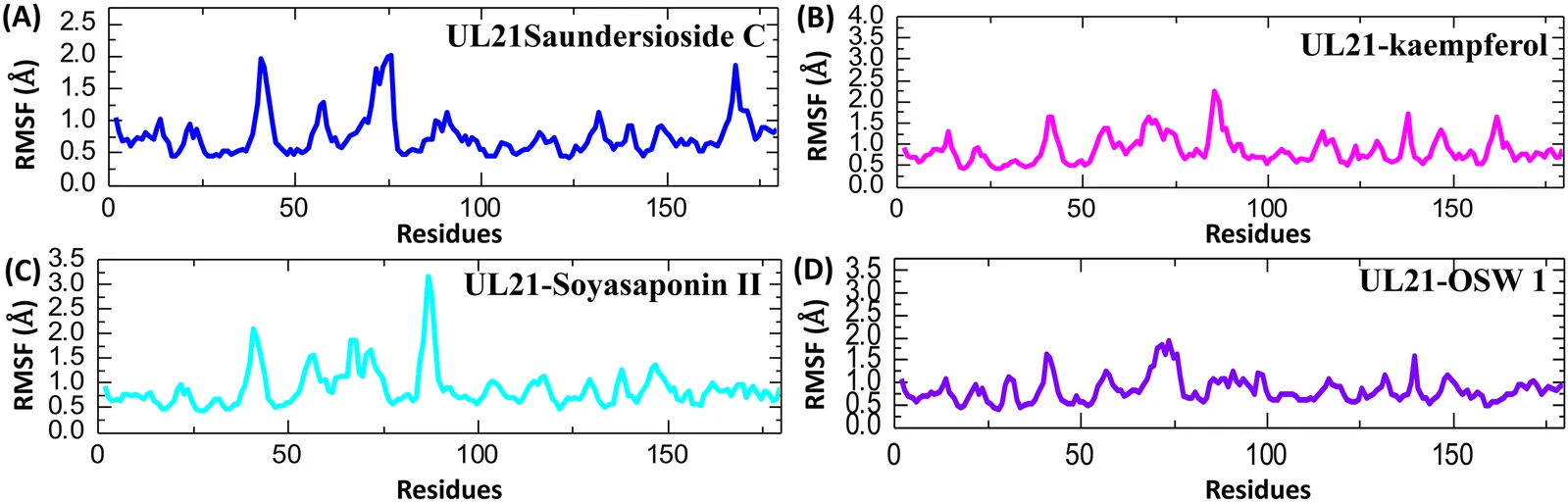
Residual fluctuation analysis of UL21-ligand complexes after molecular dynamics simulation was carried out using RMSF profiles. (A) Residue flexibility pattern of the UL21-Saundersioside C complex. (B) RMSF profile corresponding to the UL21-Kaempferol complex. (C) Amino acid mobility analysis of the UL21-Soyasaponin II complex. (D) RMSF-based flexibility assessment of the UL21-OSW-1 complex throughout the simulation period.

Root Mean Square Fluctuation (RMSF) is a key metric in molecular dynamics simulations used to evaluate the residue-wise flexibility of a protein during the simulation period. Unlike RMSD, which measures global structural displacement, RMSF highlights localized fluctuations at specific amino acids, allowing identification of flexible loops, terminal regions, and rigid structural cores. RMSF is a key parameter in protein-ligand complex investigations since binding of a ligand could either stiffen the framework of the surrounding residues or cause localized mobility depending on the interaction type. Thus, the RMSF evaluation of the UL21-ligand complexes reveals how each of the compounds affects the dynamic behavior of the UL21 regions, which are significant for both structural stability and functional regulation. The RMSF profiles of the UL21-ligand complexes show distinct but similar fluctuation modes among the four systems, indicating that each compound differently affects the local residue flexibility of the UL21 N-terminal domain (Fig 5). In case of the UL21- Saundersioside C complex shows slightly higher fluctuations in selective regions, particularly around residues 45 and 70, yet still within acceptable ranges that indicate localized loop movement without compromising structural integrity; overall, the majority of residues remain below 1 Å, confirming stable ligand engagement (Fig 5A). A similar trend is observed in the UL21-kaempferol complex, where fluctuations remain mostly within 0.5–1.5 Å, accompanied by a pronounced peak around residue ~75–85, suggesting that this region consistently displays intrinsic mobility regardless of ligand type (Fig 5B). Furthermore, UL21-Soyasaponin II complex, most residues exhibit relatively low fluctuations (0.4–1.2 Å), with a minor peak at different positions. However, one notable peak near residue ~80 indicating a flexible loop region, while the remaining protein maintains a stable backbone (Figure 5C). Similarly, the UL21-OSW-1 complex maintains a comparable fluctuation pattern, with small peaks distributed across the sequence but no extreme variations, indicating that OSW-1 binding does not induce significant destabilization (Fig 5D). Across all complexes, the fluctuation peaks correspond to naturally flexible surface loops, while the core structural residues remain stable, confirming that ligand binding preserves the global stability of UL21 while allowing normal dynamic behavior in peripheral regions.

### Structural compactness analysis of lead compounds-UL21 complexes

**Fig 6:**
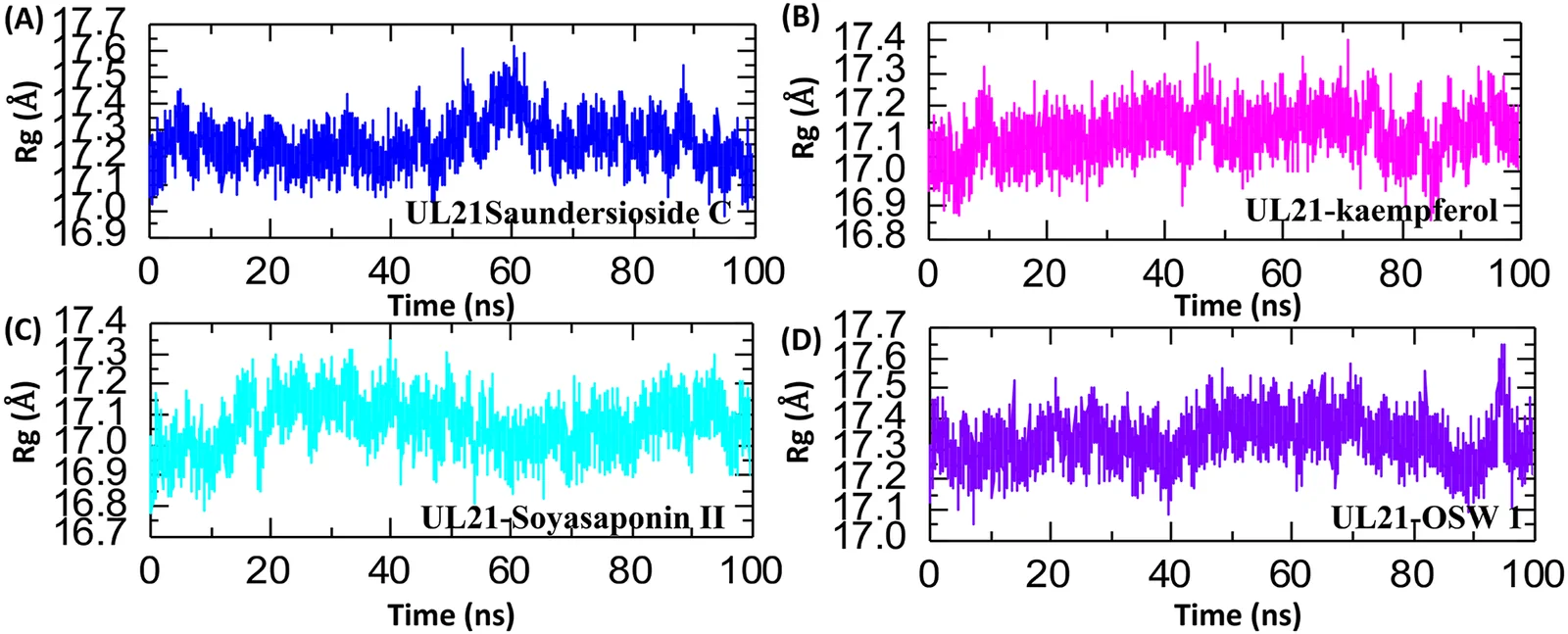
Post-simulation trajectory analysis of UL21-ligand complexes based on radius of gyration (Rg) was performed to evaluate structural compactness over the simulation time. (A) Compactness profile of the UL21-Saundersioside C complex. (B) Rg variation pattern of the UL21-Kaempferol complex. (C) Structural compactness analysis of the UL21-Soyasaponin II complex. (D) Rg variation pattern of the UL21-OSW-1 complex.

The radius of gyration (Rg) serves as an essential structural characteristic in molecular dynamics simulations and helps to evaluate the total compactness and global stability of protein-ligand complexes, where stable or slightly varying Rg values denote that the structural maintenance was during the simulation. In the present study, the Rg profiles of the UL21-ligand complexes remained consistently stable over the 100 ns trajectories, highlighting that ligand binding did not induce any major conformational expansion of the UL21 protein (Fig 6). In case of UL21-Saundersioside C complex the system showed narrow fluctuation around an average of ~17.2 Å with only subtle variations, further confirming stable structural packing and minimal long-range conformational drift (Fig 6A). In a similar manner, the Rg value for UL21-kaempferol complex showed a pattern which was similar to that of the first complexes; however, in this case, the Rg value increased up to 40 ns and then there were no significant fluctuations, indicating that the protein had retained its native global structure throughout the simulation (Fig 6B). Furthermore, the UL21-Soyasaponin II complex showed Rg values ranging from 16.9 to 17.3 Å, indicating that the protein maintains consistent structural compactness during ligand binding (Fig 6C). For UL21-OSW-1, the Rg varied from ~17.1 to 17.6 Å, indicating preserved structural compactness and further confirmed the strong stabilization seen in the docking and RMSD analyses. (Fig 6D). All four complexes show a consistent pattern of stable Rg and RMSF trajectories, indicating maintained structural compactness and controlled residue-level fluctuations of UL21, consistent with the RMSD analysis.

### Post-simulation hydrogen bond analysis of lead compounds-UL21 complexes

**Fig 7:**
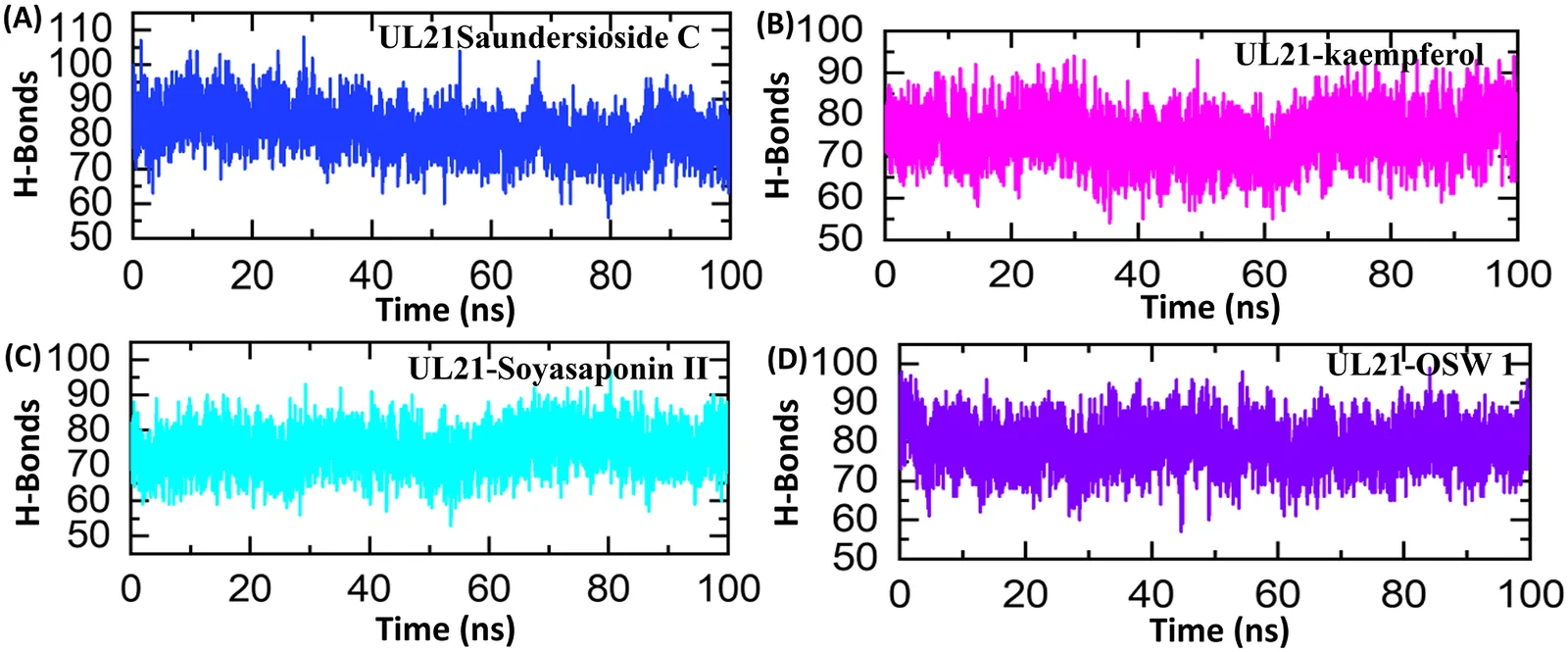
Analysis of post-simulation total hydrogen-bonding network of the solvated system of each complex. (A) Illustrates the hydrogen bonds trajectory of UL21-Saundersioside C complex. (B) Time-dependent hydrogen bonding pattern in the UL21-Kaempferol complex. (C) Illustrates the hydrogen bonds trajectory of UL21-Soyasaponin II complex. (D) Hydrogen bond stability analysis of the UL21-OSW-1 complex throughout the simulation period.

In the course of molecular dynamics simulations, the H-bonding phenomenon is a core aspect that helps in the stabilization of the protein-ligand interactions, since the existence of H-bonds for a long period is an indication of the strong interactions between molecules and is one of the major factors for the overall binding energy and structural stability. During the current investigation, the UL21-ligand combinations showed very high levels of hydrogen bonds during the entire 100 ns simulations, thus confirming the dynamic stability that was already detected in the previous analyses of the RMSD, RMSF, and Rg parameters (Fig 7). The UL21-Saundersioside C complex maintained between 70 and 100 H-bonds, with dense and frequent fluctuations indicative of a highly interactive binding interface (Fig 7A). Similarly, the UL21-kaempferol complex showed a slightly lower range (60−90 H-bonds), with fewer abrupt deviations, suggesting a more uniform and stable hydrogen-bonding pattern that aligns with the compound’s strong docking affinity (Fig 7B). Furthermore, the UL21-Soyasaponin II and UL21-OSW-1 complexes displayed a similar pattern of H-bond distribution, with moderate fluctuations representing flexible yet persistent intermolecular interactions that support long-term complex stability (Fig 7C, 7D). Overall, the high frequency and stability of hydrogen bonds across all four systems confirm that each ligand forms persistent contacts with UL21, further validating their potential as effective inhibitors capable of disrupting UL21-mediated immune evasion.

### Binding free energy (TBE) calculation of lead compounds-UL21 complexes

Binding free energy analysis acknowledges the dynamic behavior of protein-ligand complexes during the simulations and hence provides a more accurate evaluation of their interaction [56]. MM/GBSA and MM/PBSA methods disassemble the overall interaction energy into different contributions, letting one get a better insight into the stabilization factors of each complex, in contrast to static docking scores. The methods not only validate the docking results but also aid in selecting the most potent candidates for subsequent optimization [57]. Consequently, MM/GBSA and MM/PBSA analyses were performed to estimate the binding affinities of the UL21-ligand complexes. The results from both methods demonstrated a consistent binding trend among the four studied compounds, although differences in the absolute binding energies were observed due to the distinct solvation models employed in MM/GBSA and MM/PBSA calculations. In both analyses, kaempferol exhibited the strongest binding affinity toward UL21, with ∆G_total values of −40.9 kcal/mol (MM/GBSA) and −33.9 kcal/mol (MM/PBSA). Its strong interaction was mainly driven by highly favorable van der Waals interactions (ΔE_vdw = −52.1 kcal/mol) along with relatively low solvation penalties, indicating stable and energetically favorable complex formation. Soyasaponin II showed the second strongest binding affinity in the MM/GBSA analysis, with a ∆G_total value of −37.5 kcal/mol, primarily due to highly favorable van der Waals (ΔE_vdw = −52.2 kcal/mol) and electrostatic interactions (ΔE_ele = −20.7 kcal/mol). However, these favorable interactions were partially offset by substantial polar solvation energies (E_GB = 41.4 kcal/mol in MM/GBSA and E_PB = 25.0 kcal/mol in MM/PBSA), which reduced the overall binding strength. Saundersioside C also demonstrated stable binding with ∆G_total values of −30.8 kcal/mol (MM/GBSA) and −23.9 kcal/mol (MM/PBSA). Its binding profile was mainly supported by favorable van der Waals interactions (ΔE_vdw = −40.1 kcal/mol) combined with moderate solvation contributions, indicating a balanced interaction pattern within the UL21 binding pocket. In contrast, OSW-1 consistently displayed the weakest binding affinity in both methods, with ∆G_total values of −17.9 kcal/mol (MM/GBSA) and −15.5 kcal/mol (MM/PBSA). This weaker affinity was mainly associated with its extremely high positive electrostatic energy (ΔE_ele = 220.3 kcal/mol), which created an unfavorable gas-phase energy profile despite partial compensation from highly favorable polar solvation energies (E_GB = −207.5 kcal/mol and E_PB = −206.1 kcal/mol). Overall, MM/PBSA predicted slightly less favorable binding energies than MM/GBSA for all complexes due to the stronger desolvation penalties imposed by the Poisson-Boltzmann solvation model. Nevertheless, both methods consistently ranked the ligands in the same order of binding affinity: kaempferol > soyasaponin II > Saundersioside C > OSW-1, highlighting kaempferol as the most promising natural inhibitor of UL21. The detailed MM/GBSA and MM/PBSA binding energy values are presented in **Table 2**.

**Table 2:** List of binding free energies calculated by using the MM/GBSA approach. All values are expressed in kcal/mol.

MM/GBSA				
Parameters	UL21- Saundersioside C	UL21-kaempferol	UL21-Soyasaponin II	UL21-OSW-1
ΔEvdw	−40.1	−52.1	−52.2	−27.09
ΔEele	−0.5	1.8	−20.7	220.3
EGB	14.9	15.5	41.4	−207.5
ESURF	−5.0	−6.1	−6.0	−3.7
Delta G Gas	−40.6	−50.3	−72.9	193.2
Delta G Solv	9.9	9.4	35.4	−211.2
**∆G total**	−30.8	−40.9	−37.5	−17.9
**MM/PBSA**				
**Parameters**	**UL21- Saundersioside C**	**UL21-kaempferol**	**UL21-Soyasaponin II**	**UL21-OSW-1**
ΔEvdw	−40.1	−52.1	−52.2	−27.09
ΔEele	−0.5	1.8	−20.7	220.3
EPB	11.4	9.3	25	−206.1
ENPOLAR	−25	−40.8	−30.6	−29.9
EDISPER	30.3	47.9	54.8	27.1
Delta G Gas	−40.6	−50.3	−72.9	193.2
Delta G Solv	42.7	46.4	73.3	−186.9
**∆G total**	−23.7	−33.9	−23.8	−15.5

### PCA and FEL analysis of lead compounds-UL21 complexes

**Fig 8:**
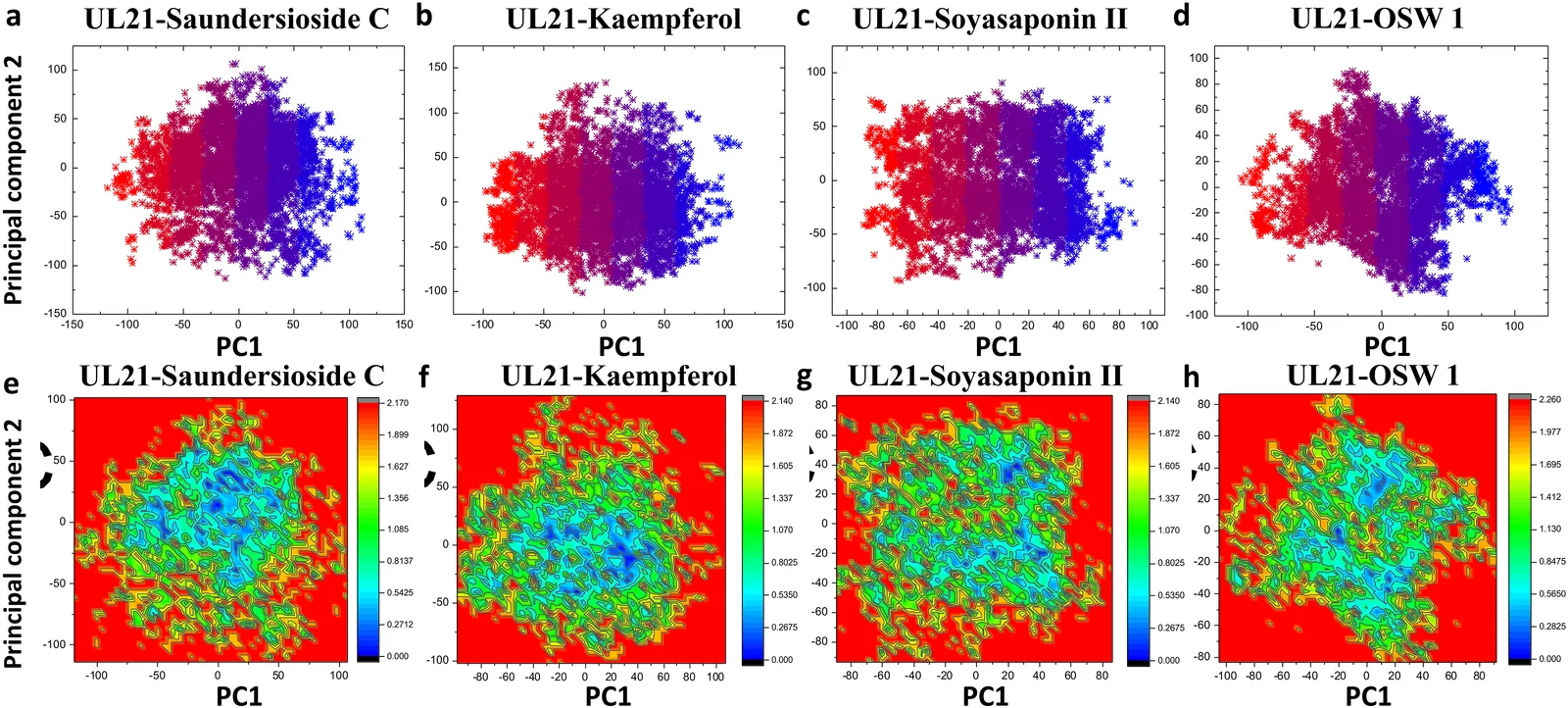
Principal component analysis (PCA) and free energy landscape (FEL) analysis of UL21-ligand complexes. (A-D) Showing PCA projections of UL21 bound to the selected lead compounds. (E-H) Represents FEL plots of the corresponding UL21-ligand complexes.

The PCA and FEL analyses collectively illustrate the conformational dynamics and stability of the UL21 protein when complexed with the selected lead compounds Saundersioside C, Kaempferol, Soyasaponin II, and OSW-1. The PCA plots reveal that all the complexes have clustered and overlapping trajectories within a limited conformational space, which signifies that the binding of the ligand hinders UL21 motion and supports stable structural dynamics throughout the simulation. Among the different ligands, the two compounds, Saundersioside C and Kaempferol (Fig 8A, 8B), are at the one end of the principal component axes (PC1 and PC2) showing the smallest degree of structural fluctuations and the strongest stabilization of UL21; the latter being in the case of Soyasaponin II and OSW-1, which display somewhat wider distribution patterns (Fig 8C, 8D). The FEL graphs additionally corroborate these findings, all complexes have clear low-energy basins (blue and cyan regions), which correspond to the most favorable conformational states for them; however, Saundeioside C and Kaempferol show deeper and more central minima, which means that these ligands keep UL21 in more favorable conformations (Fig 8E and 8F). Soyasaponin II and OSW-1 reveal a bit more spread-out minima, which is a sign of greater conformational transitions (Fig 8G, 8H). To sum up, the joint PCA and FEL findings show that all four compounds during the simulation still ensure the structural integrity of UL21, with Saundersioside C and Kaempferol giving the strongest stabilizing effect.

### Pharmacokinetics analysis of selected compounds

The ADMET analysis of the selected compounds Saundersioside C, kaempferol 3,7,4′-tri-O-β-glucoside, Soyasaponin II, and OSW-1 provides a comprehensive insight into their pharmacokinetic behavior and safety profiles. In terms of absorption, all compounds exhibit moderate water solubility (Log S ranging from −2.76 to −3.71), indicating acceptable dissolution characteristics, although Saundersioside C and kaempferol derivatives show lower Caco-2 permeability and poor human intestinal absorption, suggesting limited oral bioavailability. In contrast, OSW-1 demonstrates relatively better permeability and high intestinal absorption, making it more favorable for oral delivery. Regarding distribution, all compounds show low volume of distribution (VDss), implying limited tissue penetration, while their low blood-brain barrier (BBB) and CNS permeability values indicate minimal likelihood of central nervous system exposure, which is advantageous for reducing CNS-related side effects. From a metabolic perspective, none of the compounds act as substrates of CYP2D6, reducing variability due to genetic polymorphism, while Saundersioside C and OSW-1 are substrates of CYP3A4, suggesting possible metabolism through this major enzyme; importantly, only OSW-1 shows CYP3A4 inhibitory activity, which may raise concerns about drug-drug interactions. In terms of excretion, the total clearance values vary, with Saundersioside C showing relatively higher clearance, indicating faster elimination, whereas others demonstrate moderate clearance rates. Toxicity predictions are particularly favorable, as all compounds are non-AMES toxic, non-hepatotoxic, and non-sensitizing to skin, highlighting a strong safety profile (S1 Table). Overall, while most compounds exhibit good safety and metabolic stability, OSW-1 stands out for its superior absorption characteristics, whereas the others may require formulation strategies to improve bioavailability.

## Conclusion

This study provides a comprehensive computational framework to identify and validate natural-product-based inhibitors targeting the HSV-1 UL21 protein, a critical mediator of viral immune evasion. Through integrated virtual screening, induced-fit docking, molecular dynamics simulations, and binding free energy calculations, four lead compounds Saundersioside C, Kaempferol 3,7,4′-tri-O-β-glucoside, Soyasaponin II, and OSW-1 were identified with strong binding affinity and stable interaction profiles. Among these, kaempferol 3,7,4′-tri-O-β-glucoside emerged as the most promising candidate, demonstrating the lowest binding free energy and consistent structural stability across all simulation parameters, including RMSD, RMSF, Rg, and hydrogen bonding. Importantly, all lead compounds maintained stable conformations within the UL21 binding pocket and preserved the structural integrity of the protein, suggesting their potential to effectively disrupt UL21-mediated suppression of the cGAS-STING immune pathway. The agreement between docking scores, dynamic stability analyses, and MM/GBSA/MM/PBSA results strengthens the reliability of these findings. Notably, this study identifies UL21 as a tractable and underexplored antiviral target and provides the first computational evidence that natural-product scaffolds can potentially disrupt its immune-evasion function. Among the identified compounds, the kaempferol glycoside demonstrated the most favorable binding characteristics and dynamic stability, highlighting its promise as a lead scaffold for future antiviral drug development. Overall, this work not only identifies potent natural compounds with antiviral potential but also establishes a strong foundation for future experimental validation and rational optimization of these molecules.

## Supporting information

S1 File ADMET properties analysis of selected compounds. (DOCX)

S2 File Supporting files. (ZIP)
