# Supplementary material for: Mechanistic inhibition of herpes simplex virus-1 UL21 immune-evasion function by natural-product scaffolds: A multi-tier docking, dynamics, and energetic profiling approach
Source: PLoS One. 2026 Sep 16;21(9):e0355499. doi: 10.1371/journal.pone.0355499 (PMC13581210; doi:10.1371/journal.pone.0355499)
Supplement: Supplementary file 1 [file pone.0355499.s001.docx]

**Suplementary information**

**Supplementary Table S1. ADMET properties analysis of selected compounds**

| **Properties** | **Saundersioside C** | **kaempferol** | **Soyasaponin II** | **OSW-1** |
| --- | --- | --- | --- | --- |
| **Absorption** | | | | |
| Water solubility  Log S | -2.95 | -2.766 | -2.887 | -3.718 |
| Caco-2 permeability  x 10−6 | -1.054 | -1.031 | -0.413 | 0.383 |
| Human Intestinal absorption (%) | Low | No | No | High |
| **Distribution** | | | | |
| VDss (human) | -0.647 | -0.399 | -0.435 | -0.249 |
| BBB  permeability | -2.075 | -2.777 | -1.293 | -1.952 |
| CNS  permeability | -5.02 | -5.975 | -4.106 | -4.045 |
| **Metabolism** | | | | |
| CYP2D6 substrate | No | No | No | No |
| CYP3A4 substrate | Yes | No | No | Yes |
| CYP1A2 inhibitor | No | No | No | No |
| CYP2C19 inhibitor | No | No | No | No |
| CYP3A4 inhibitor | No | No | No | Yes |
| **Excretion** | | | | |
| Total Clearance | 0.519 | -0.248 | -0.028 | 0.145 |
| Renal OCT2 substrate | No | No | No | No |
| **Toxicity** | | | | |
| AMES toxicity | No | No | No | No |
| Skin sensitization | No | No | No | No |
| Hepatotoxicity | No | No | No | No |
